# Genome Sequence Conservation of Hendra Virus Isolates during Spillover to Horses, Australia

**DOI:** 10.3201/eid1611.100501

**Published:** 2010-11

**Authors:** Glenn A. Marsh, Shawn Todd, Adam Foord, Eric Hansson, Kelly Davies, Lynda Wright, Chris Morrissy, Kim Halpin, Deborah Middleton, Hume E. Field, Peter Daniels, Lin-Fa Wang

**Affiliations:** Author affiliations: Australian Animal Health Laboratory, Geelong, Victoria, Australia (G.A. Marsh, S. Todd, A. Foord, E. Hansson, K. Davies, L. Wright, C. Morrissy, K. Halpin, D. Middleton, P. Daniels, L.-F. Wang);; Australian Biosecurity Cooperative Research Centre for Emerging Infectious Diseases, Brisbane, Queensland, Australia (H.E. Field, L.-F. Wang);; Biosecurity Queensland, Brisbane (H.E. Field)

**Keywords:** Viruses, paramyxovirus, henipavirus, genome sequence, bat virus, horses, Australia, zoonoses, dispatch

## Abstract

Bat-to-horse transmission of Hendra virus has occurred at least 14 times. Although clinical signs in horses have differed, genome sequencing has demonstrated little variation among the isolates. Our sequencing of 5 isolates from recent Hendra virus outbreaks in horses found no correlation between sequences and time or geographic location of outbreaks.

*Hendra virus* (HeV) (family *Paramyxoviridae*, subfamily *Paramyxovirinae*, genus *Henipavirus*) was first isolated in 1994 during an outbreak of respiratory disease in horses at a stable in Hendra, a suburb of Brisbane, Queensland, Australia. During the outbreak, all horses at the affected property were tested. Thirteen horses died; 7 recovered with seroconversion, some without clinical signs; and 9 remained uninfected. A horse trainer and a stable worker also became infected, and the trainer subsequently died. In October 1995, HeV infection was diagnosed for a third person, who lived in Mackay, ≈1,000 km north of Brisbane. Retrospective analysis demonstrated that an outbreak had occurred in Mackay in August 1994, where the infected person had assisted with necropsies on 2 horses. At the time, he recovered from a mild undiagnosed infection. Fourteen months later, fatal encephalitis developed, suggesting either virus persistence or late onset of disease symptoms (*1*). Since 1994, a total of 12 outbreaks have occurred (Table 1).

**Table 1:** Hendra virus outbreaks affecting horses and humans, Australia, August 1994–May 2010*

Date	Location	Horses, no. cases	Humans, no. cases/no. deaths
1994 Aug	Mackay, QLD	2	1/1
1994 Sep	Hendra, QLD	20	2/1
1999 Jan	Trinity Beach, QLD	1	0/0
2004 Oct	Gordonvale, QLD	1	1/0
2004 Dec	Townsville, QLD	1	0/0
2006 Jun	Peachester, QLD	1	0/0
2006 Oct	Murwillumbah, NSW	1	0/0
2007 Jun	Peachester, QLD	1	0/0
2007 Jul	Clifton Beach, QLD	1	0/0
2008 Jul	Redlands, QLD	5	2/1
2008 Jul	Proserpine, QLD	3	0/0
2009 Jul	Rockhampton, QLD	3	1/1
2009 Sep	Bowen, QLD	3	0/0
2010 May	Tewantin, QLD	1	0/0
Total		44	7/4

*QLD, Queensland; NSW, New South Wales.

Serologic evidence identified flying foxes (genus *Pteropus*) as the likely reservoir host, and HeV was subsequently isolated from 2 species of pteropid bats (*2*). Serologic evidence of HeV in bats has been demonstrated along the east coast of Australia to Melbourne and west across northern Australia to Darwin. Seroprevalence can be as high as 25% (*3*). In recent years, the regularity of spillover events has increased. Increased monitoring of bat population sizes, virus in bat populations, and virus characterization is necessary to better learn about trigger(s) for spillover events.

*Nipah virus* (NiV) is the only other known species within the genus *Henipavirus*. NiV was first identified during a major outbreak of diseases in pigs and humans in peninsular Malaysia during 1998–99. NiV reemerged in Bangladesh in 2001, with recurrence resulting in human infection almost annually in Bangladesh and India (*4*). Serologic evidence of NiV or NiV-related viruses has been demonstrated in bats in Thailand (*5*), Indonesia (*6*), People’s Republic of China (*7*), Madagascar (*8*), and west Africa (*9*); virus has been isolated from flying foxes in Malaysia (*10*) and Cambodia (*11*).

A major characteristic of henipavirus infections is their systemic spread, with evidence of infection in multiple organ systems. HeV infection in horses typically produces an acute, febrile respiratory disease (*12*) with a high case-fatality rate. The 2008 Redlands outbreak was the largest in horses since the first identified outbreak in 1994. During this outbreak, infected horses showed atypical signs of HeV infections, with clinical features of a more neurologic nature (*13*). Before the outbreak was attributed to HeV, 2 persons became infected, resulting in 1 death and the potential exposure of >50 persons. The reason for the altered clinical picture during this spillover event is unknown.

## The Study

Little is known about the genetic variation of henipaviruses because few sequences are available; most sequences came from the NiV outbreak in Malaysia and Singapore. From the NiV outbreak in Malaysia, sequences were obtained for isolates from 4 humans, 4 pigs, and 1 bat. One sequence is available for the original HeV isolate and 1 isolate each of NiV from Bangladesh and India. A limited sequence for the *N* and *G* genes for 2 isolates from Cambodia has been deposited in GenBank.

Five additional isolates from horses were obtained from outbreaks in Murwillumbah (2006), Peachester (2007), Clifton Beach (2007), Redlands (2008), and Proserpine (2008). These isolates provided us with a unique opportunity to examine HeV sequence variation over time and geographic separations.

Analysis of the sequences demonstrated the extreme conservation at the genome and protein levels. All isolates had the identical genome length of 18,234 nt, with the sequence variation across the full genome being <1%. All open reading frames (ORFs) were the same length as those of the original HeV isolate (Hendra virus/Australia/horse/1994/Hendra). Minor nucleotide changes were seen in the ORFs and the noncoding regions. The *N* ORF had the highest percentage of changes in both nucleotide and amino acid variation (Table 2). The number of nucleotide changes was lower than those in other RNA viruses; for example, the *N* gene of measles virus showed up to 7% variation in nucleotide sequence (*14*). The predicted size of each of the *P* gene products (V, W, and C) was conserved in all isolates. The small basic (SB) protein ORF identified in the original HeV isolate of HeV, but not seen in any of the NiV strains, was present in all isolates with an identical length of 65 aa. The variation in this ORF was higher than that for other ORFs, with 0–6% aa variation. Although SB is in an alternate frame within the *P* gene, the variation was much higher than that of the *C* ORF, which is also in an alternate ORF.

**Table 2:** Nucleotide and amino acid residue changes in recent Hendra virus isolates compared with the original isolate, Australia

Isolate	Full genome variation (%)	Open reading frame (length in nt)																
		*N* (1,599)			*P* (2,124)			*M* (1,059)			*F* (1,641)			*G* (1,815)			*L* (6,735)	
		nt	aa		nt	aa		nt	aa		nt	aa		nt	aa		nt	aa
Hendra virus/Australia/horse/2006/Murwillumbah (GenBank accession no. HM044318)	171 (0.94)	16	6		5	4		6	2		2	1		4	2		39	10
Hendra virus/Australia/horse/2007/Peachester (GenBank accession no. HM044319)	87 (0.47)	2	1		13	4		5	1		10	1		12	2		57	11
Hendra virus/Australia/horse/2007/Clifton Beach (GenBank accession no. HM044321)	139 (0.76)	12	4		6	3		5	1		11	1		5	0		48	11
Hendra virus/Australia/horse/2008/Redlands (GenBank accession no. HM044317)	186 (1.02)	16	4		14	2		4	1		13	1		11	0		59	12
Hendra virus/Australia/horse/2008/Proserpine (GenBank accession no. HM044320)	183 (1.00)	17	4		15	4		7	2		9	1		11	1		64	11

Phylogenetic analysis was performed by using both DNA and amino acid sequences for individual genes and for the complete genome. The multiple branches of the phylogenetic tree, particularly for the *N* gene (Figure), suggest that all these isolates branched from an ancestor earlier than 1994 when the first identified outbreak occurred. For example, the 2006 isolate from Murwillumbah shows greatest similarity to the 1994 isolate, whereas the other isolates are in a separate branch on the tree.

**Figure:**
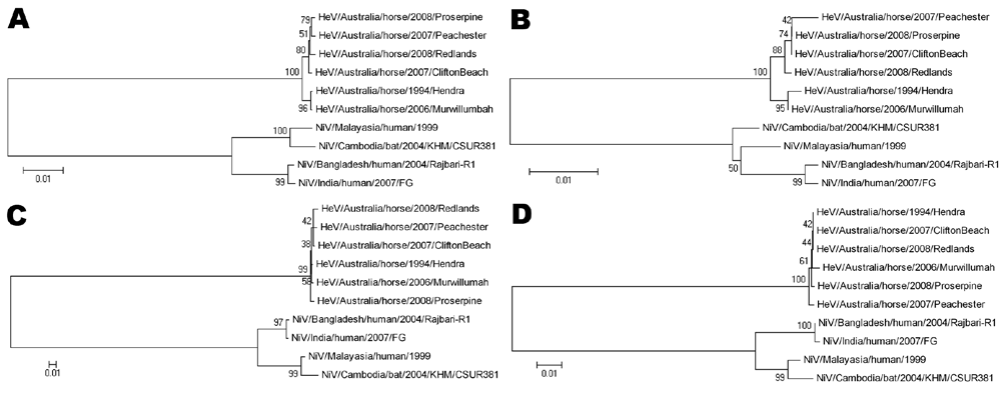
Phylogenetic trees based on the N open reading frame (ORF) (A, B) and the G ORF (C, D), with DNA sequences used for A and C and amino acid sequences for B and D. All sequences were compared with the reference sequences for each of the known henipavirus strains; Hendra virus/Australia/horse/1994/Hendra (GenBank accession no. AF017149), Nipah virus/Malaysia/human/1999 (GenBank accession no. AF212302), Nipah virus/Bangladesh/human/2004/Rajbari, R1 (GenBank accession no. AY988601), Nipah virus/Cambodia/bat/2004/KHM/CSUR381 (GenBank accession no. AY858110 [*N* ORF] and AY858111 [*G* ORF]) and Nipah virus/India/human/2007/GF (GenBank accession no. FJ513078). Phylogenetic trees were constructed by using the neighbor-joining algorithm in the MEGA4 software package (*15*). Scale bars represent substitutions per site. HeV, Hendra virus; NiV, Nipah virus.

The data reported here are consistent with each of these individual spillover events that occurred after exposure to viral variants coming from a large pool of quasispecies in the bat population in Australia. This genetic conservation in HeV isolates may suggest that HeV is genetically stable in the reservoir bat population, although because of the lack of HeV isolates from bats, the possibility cannot be excluded that this genetic similarity resulted from selection both for variants that can infect horses and selection within the infected horse. Further analysis of this sequence data and future sequence information will help with understanding of differences in the clinical picture and may provide evidence to explain the apparent increase in regularity of recent spillover events.

## Conclusions

Our results demonstrated that HeV isolates from horses are genetically similar, with variation at both the nucleotide and amino acid levels, uniformly small, and <1% across the 18.2-kb genome. Furthermore, data show that different outbreaks resulted from independent spillover events from a pool of HeV quasispecies in the fruit bat populations in Australia. The genetic relatedness of HeV isolates from horses is not correlated with the time the corresponding HeV outbreaks occurred. The presence of SB ORF in all HeV isolates, but not in NiV isolates, warrants further functional analysis of this intriguing putative protein.
